# Socioeconomic indicators of heat-related health risk supplemented with remotely sensed data

**DOI:** 10.1186/1476-072X-8-57

**Published:** 2009-10-16

**Authors:** Daniel P Johnson, Jeffrey S Wilson, George C Luber

**Affiliations:** 1 Department of Geography, School of Liberal Arts, Indiana University - Purdue University Indianapolis, Indiana, USA; 2 National Center for Environmental Health, Centers for Disease Control and Prevention, Atlanta, Georgia, USA

## Abstract

**Background:**

Extreme heat events are the number one cause of weather-related fatalities in the United States. The current system of alert for extreme heat events does not take into account intra-urban spatial variation in risk. The purpose of this study is to evaluate a potential method to improve spatial delineation of risk from extreme heat events in urban environments by integrating sociodemographic risk factors with estimates of land surface temperature derived from thermal remote sensing data.

**Results:**

Comparison of logistic regression models indicates that supplementing known sociodemographic risk factors with remote sensing estimates of land surface temperature improves the delineation of intra-urban variations in risk from extreme heat events.

**Conclusion:**

Thermal remote sensing data can be utilized to improve understanding of intra-urban variations in risk from extreme heat. The refinement of current risk assessment systems could increase the likelihood of survival during extreme heat events and assist emergency personnel in the delivery of vital resources during such disasters.

## Background

The impact of climate change on human health is a major concern for the global public health community [1-5]. Health outcomes expected to be impacted by climate change include, but are not limited to, asthma, heart disease, infectious diseases and heat-related illnesses [6-9]. In North America, extreme heat events (EHEs) are the number one cause of weather-related mortality [10]. This is likely the case for countries across the world, although it is difficult to prove due to lack of health surveillance data [11]. Climate models project year-round temperatures across North America for the first half of the 21^st ^century will warm approximately 1 to 3°C [12], increasing the magnitude and duration of EHEs in portions of the U.S. where they already occur [13]. Late in the 21^st ^century, projected annual warming is likely to be 2 to 3°C across the western, southern, and eastern continental margins, but more than 5°C at higher latitudes[13], where many U.S. urban areas that have been affected by lethal heat waves are located.

Despite the projections of a warming climate and an increase in EHE frequency and intensity, there is a lack of public recognition involving the hazard of extreme heat exposure. U.S. metropolitan areas generally lack preparedness measures such as heat wave response plans [14-16]. Much of the problem lies in the fact that heat waves are silent killers that do not leave a trail of physical destruction in their wake. Like other natural disasters they are sporadic phenomena, but unlike hurricanes or tornadoes, heat waves do not leave lasting reminders of physical devastation.

Epidemiologic studies indicate that individuals at higher risk of adverse health effects from extreme heat exposure include the elderly, the urban poor, those living alone, and persons who do not have access to air conditioning [17-20]. In addition, persons with chronic mental disorders, pre-existing medical conditions (obesity, cardiovascular, neurological and psychiatric diseases) are at elevated risk. Medications that interfere with salt and water balance, such as diuretics, anticholergic agents, and tranquilizers that impair sweating, also increase risk.

There has been a renewed emphasis on the relationship between place and human health. Recent reviews have recommended increased integration of spatial information in health behaviour and health outcomes research to develop a more comprehensive understanding of place-based effects, as well as new analytical approaches [21,22]. Census data provide information about the spatial distribution of some sociodemographic (population-based) characteristics associated with vulnerability to EHEs at multiple levels of aggregation (county, census tract, census block group). Even with the availability of population indicators from census data, surveillance and alert for heat-related conditions is currently only conducted at a regional or county level [19,23-25]. This resolution of surveillance lacks sufficient spatial detail to account for intra-urban variability in risk. Methods that provide more spatially specific information may better inform planning and intervention in areas where increased prevalence of heat-related illness is likely to occur.

Previous studies of EHE risk factors suggest mapping sociodemographic variables (i.e. vulnerability, population density) from census data to provide indication of the spatial variation in vulnerability [26-30]. However, this approach does not account for physical environment variables that may contribute to increased risk from EHEs. For example, vulnerable residents living in an area of low environmental heat load may be less at risk than a group living in an area of high heat load. Accounting for the coincident relationships between both social and physical environmental factors in assessing risk from EHEs may support improved planning and intervention strategies.

The environmental heat load in urban areas is partially indicated by the urban heat island (UHI) effect. The UHI is the observed difference between the rural and urban temperature gradient [31]. Typically, surface temperatures in urban areas are higher than rural locations. This phenomenon may have an exacerbating effect during heat waves and potentially contributes to heat-related death [32,33]. Moreover, the UHI effect is spatially dynamic, consisting of differing areas of intensity within the city [34]. The UHI shows strong seasonal fluctuations in its diurnal intensity but the temperature extremes between the urban and rural areas are most pronounced during the day in the summer months [35,36]. However, the nocturnal UHI additionally shows drastic temperature disparities with the contiguous rural space. These intensity levels are strongly associated with the land cover types [37-40]. It is thought that a model incorporating surface temperature variations with socioeconomic indicators of heat-related vulnerability may yield a more robust predictor of risk than one accounting for the socioeconomic indicators alone.

## Results

All models developed are shown in table 1. The model predicting heat-related mortality using only sociodemographic count variables and total population (model 1), was found not significant using the Hosmer-Lemeshow (H-L) test. It is important to note that this particular model produced a sensitivity and specificity of .79. However, it is excluded from discussion due to its apparent lack of fit with the original variables.

**Table 1:** LOGITS, Odds Ratios and level of significance for the variables included in the models

**Models**	***B***	**Odds Ratio**	***p***
**Model 1 - Socioeconomic Counts**			
Hosmer-lemeshow Test p < .05 (not significant)			
Poverty	0.601	1.823	0.0001
Age 65 up in Poverty	0.666	1.947	0.0001

**Model 2 - Thermal Data**			
Hosmer-lemeshow Test p >.05 (significant)			
Maximum LST	-1.045	0.352	0.002
Mean LST	1.797	6.034	0.001
Range LST	0.66	1.935	0.0001

**Model 3 - Thermal Data and Socioeconomic Counts**			
Hosmer-lemeshow Test p > .05 (significant)			
Max LST	-1.186	0.305	0.002
Range LST	0.706	2.026	0.001
Mean LST	1.41	4.095	0.0001
Age 65 up in Poverty	0.639	1.894	0.0001
Poverty	0.521	1.684	0.004

**Model 4 - Socioeconomic Rates**			
Hosmer-lemeshow Test p > .05 (significant)			
Low Education Rate	0.426	1.531	0.001
African-American Rate	0.505	1.657	0.0001

**Model 5 - Thermal Data and Socioeconomic Rates**			
Hosmer-lemeshow Test p > .05 (significant)			
Max LST	-0.999	0.368	0.002
Range LST	0.715	2.044	0.004
Mean LST	1.709	5.523	0.0001
African-American Rate	0.396	1.486	0.0001

Model 2 (attributes shown in table 1) included only land surface temperature (LST) variables extracted from Landsat TM data. Maximum, mean and range of LST were found to be statistically significant variables and the overall model was significant using the Hosmer-Lemeshow test. The ROC examination shows a .72 area under the curve, but it is not statistically different from using model 4, the sociodemographic rate data (the standard). Examining the thermal variables in this model suggests that for each unit increase in the mean LST, risk of death increases by a factor of 6. The odds ratio of .352 for maximum LST indicates that for each unit decrease in maximum LST the odds of death increase by a factor of 2.84. As the maximum LST value for each census tract increases, the probability of death decreases; but not to the same degree as it increases with increased mean LST. This is somewhat counterintuitive but if the maximum LST is removed, leaving only the mean and the range, the sensitivity and specificity is degraded to .68. A standard unit increase in the range LST raised the probability of death by a factor of 1.94.

**Figure 1:**
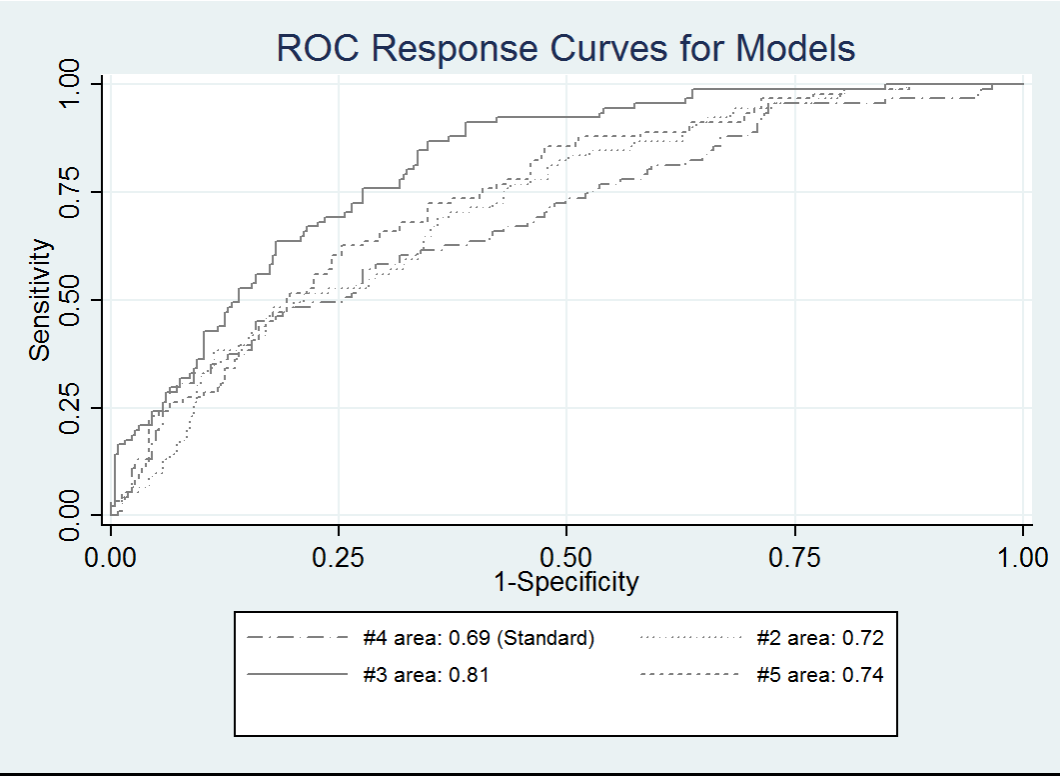
**Comparison of ROC curves**.

Model 3 assimilated LST data with the sociodemographic count and total population data. Results were significant as determined by the Hosmer-Lemeshow test. Age 65+ in poverty and total population in poverty were the only sociodemographic variables found to be significant (see table 1). Maximum, mean and range were significant LST variables. The probability of heat-related death increased by a factor of 1.89 as the count of age 65+in poverty increased by one standard unit. Similar to model 1, the odds ratio for poverty counts was 1.68. Maximum, mean, and range of LST were comparable to results observed in model 2. The inverse relation with maximum LST is still present. If maximum LST is removed the model is degraded to .79 in sensitivity and specificity as compared to .81 (2) when maximum LST is included (table 2; figure 1); this is still significantly different from the standard. Overall, model 3 performed the best in predicting heat-related mortality.

**Table 2:** ROC comparison using Model 4 (socioeconomic rates) as the gold standard

**ROC Curve Comparison against the Standard**		
**Model**	**Curve Area**	**P**

#1	0.7864	Not significant using H-L Test
#2	0.7174	0.4543
#3	0.8134	0.0001
#5	0.7407	0.0583

Model 4 included only socioeconomic rate and population density variables. Low education rate and African-American rate emerged as significant predictors, with odds ratios of 1.53 and 1.68, respectively (table 1). This model, due to the frequent usage of socioeconomic rate data in risk assessment, was used as the standard against which other models were tested. The ROC curve (table 2; figure 1) for this model is .69; indicating specificity and sensitivity. This model is significantly different from a random classification (.50) but would indicate a reasonably ineffective model for this event. The odds ratios suggest that as low education rate and African-American rate increase by a unit of 1, risk would increase by a factor 1.53 and 1.66 respectively. This finding is also supported by previous studies suggesting low education is a risk factor in heat-related death as well as other urban health problems [41-43].

Model 5 (table 1) supplemented the sociodemographic rate and population density data with LST. Low education rate was not statistically significant when LST data were included. The African-American rate produced an odds ratio of 1.49. Results for maximum, mean and range of LST are similar to model 2 and 3. As maximum LST decreases by a standard unit, the odds of death increase by a factor of 2.72. This again is counterintuitive, but maximum LST could be acting as a cap in the logistic modelling approach. When mean LST increases by a standard unit, the odds of death increase by a factor of 5.52. Range of LST produced an odds ratio of 2.04. This particular model has a specificity and sensitivity of .74 which is not significantly different from the standard (p = .058) (table 2; figure 1).

## Discussion

**Figure 2:**
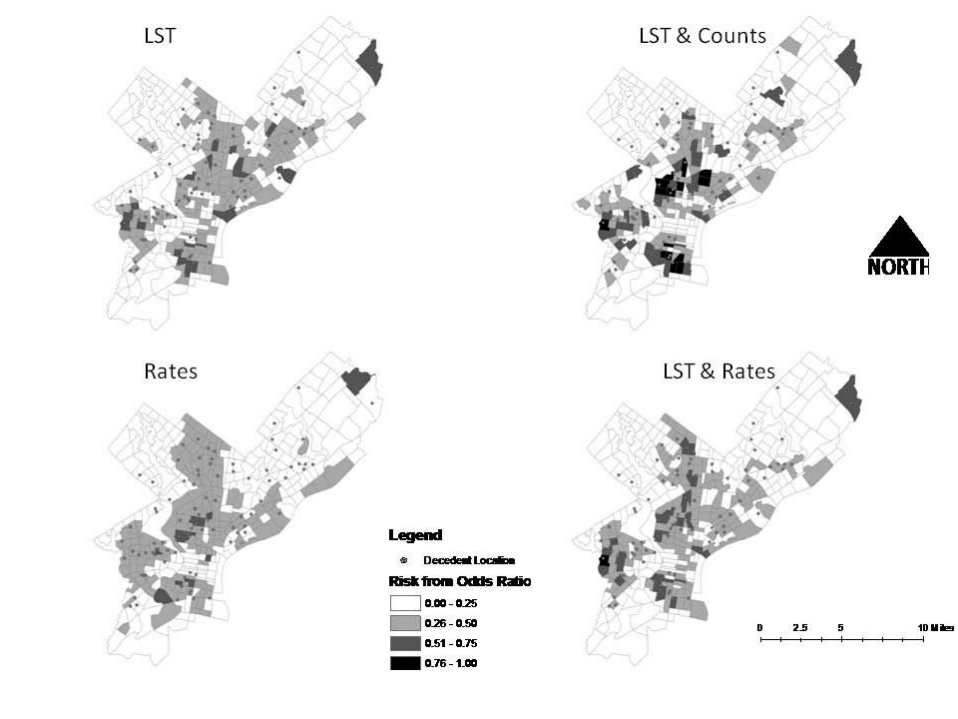
**Comparison of spatial models**.

The model incorporating only LST variables (model 2), was not statistically better than using the sociodemographic rate data alone (model 4). We did not anticipate that vulnerability models using remotely sensed LST variables alone would perform as well as one including multiple socioeconomic variables. However, figure 2 shows how the two classes of variables differ in their spatial classification. In the classification breakdowns, the LST model includes 12 census tracts in the lowest area of risk which actually included a death. The sociodemographic rate model includes 25 census tracts containing a death in this same stratification level. In this context, the sociodemographic rate model (the current standard) is outperformed by the model utilizing only remotely sensed data. Based on this finding, the LST variables appear to provide a better clue to risk than the sociodemographic rate variables tested.

Results from models 2, 4, and 5 indicate that mean LST had the highest odds ratio of the variables examined in this study. The interplay between mean LST and maximum LST appears counterintuitive; as the mean increases and the maximum decreases, the probability of death appears to be higher. Although a model not incorporating this counterintuitive association would be easier to explain, removing maximum LST degrades model performance.

**Figure 3:**
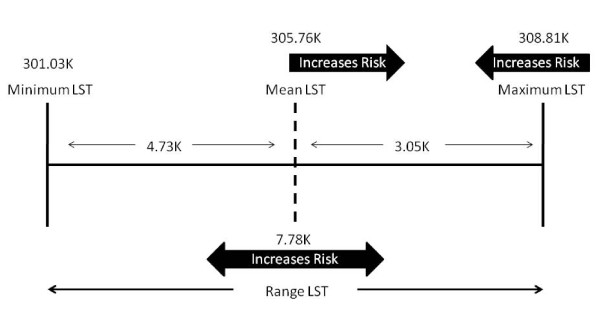
**The interplay between the LST variables**.

A possible explanation for this result is illustrated in figure 3. It is apparent and intuitive that as the mean temperature increases the likelihood of death would increase. This relationship is true when modelling only mean LST with mortality. Moreover, when the mean LST is included in any of the models, its odds ratio is the highest of all variables examined. Therefore, it is likely that the mean LST is an important component to the modelling of extreme heat vulnerability. On average, mean LST is negatively skewed; closer to the maximum than the minimum within the examined dataset. The models possibly suggest (figure 3) that the closer the mean is to the maximum, within the residential space of the census tract, the greater the risk of mortality. Previous studies have reported similar associations in temperature (average and maximum proximity) are predictive of death from extreme heat [44,45]. The inverse relationship with maximum LST may be a product of the negatively skewed nature of the mean even it is not collinear. Drawing on this analysis a possible explanation is that as the range of temperature values encountered in the neighbourhood increases, and if the mean and maximum LST are close to one another, the likelihood of death from extreme heat during this particular event would be high. Moreover, if these factors are spatially coincident with areas of high urban poverty or high proportions of African Americans the risk is even more substantial. It may be possible to test this in future studies by introducing measures of skewness and kurtosis for the thermal variables. If one encounters a negative relationship with skewness (i.e. as skewness decreases (become more negative) death increases) then the proximity of the mean with the maximum may prove to be another important component for modelling extreme heat vulnerability.

In all the models incorporating remote sensing variables, increases in the LST range variable were associated with increased risk of heat-related mortality. This result may be counter to some initial thought that lower variability of LST in warmer microclimates - suggesting the possibility of a high minimum with a high maximum - would be associated with increased risk, unless the overall temperature profile of the census tract were low. In fact, the models suggest the opposite. The increase in range suggests a broader temperature profile leading to potentially broader thermal exposures. However, the range is perhaps the lowest order measure of variability (standard deviation was not significant in any model) and when it is removed there is a degradation in sensitivity and specificity. Range LST is an important measure when describing the distribution of the high compared to the low. Therefore, as the difference between the minimum and maximum increase, coupled with the possible mean LST and maximum LST associations, the risk of death is slightly higher.

Figure 2 shows the spatial comparison between models 2-5. An issue with model 3 is that it includes 16 census tracts that had an occurrence of death in the lowest risk category (24 for the standard). However, it is more accurate in its spatial specificity when comparing the ROC curves and the spatial comparison. The most important variable in this model is the mean temperature of the census tract, which increases the risk of death by a factor of 4 for each standard unit increase.

Consistent with the overall goals of the study, remotely sensed variables proved to be important predictors of risk for heat-related mortality that improved the spatial specificity of vulnerability models compared to models that use only sociodemographic variables. In all models that included the LST variables, LST maximum, mean and range were more significant predictors than sociodemographic factors. When combined with sociodemographic factors, models incorporating LST variables were consistent with findings from previous studies that conclude older age and poverty [9,17,43] are indicators of risk and their coincident relationship with thermal characteristics [27,29] of the neighbourhood are key to indentifying risk locations.

## Conclusion

Previous studies have suggested the need for more spatial specificity in the assessment of risk from extreme heat [26-29,46]. This study builds on previous work to demonstrate a parsimonious method using logistic regression. The method employs measures of vulnerability from census variables and assimilates them with physical environment variables derived from thermal remote sensing data. The long-term goal of this application is to provide local emergency response personnel with a practical tool to better plan and prepare for heat waves by facilitating better resource allocation and tailoring of health communication messages to groups most at risk. Further examination should yield to the spatial examination of vulnerability, including the spatial arrangement of at risk groups in relation to thermal properties of the environment.

In review, heat mortality data for the Philadelphia 1993 EHE were collected from the Pennsylvania Department of Vital Records and assimilated with vulnerability and LST data. Models utilizing the sociodemographic data in the form of counts coupled with measures of LST provided the best assessment of vulnerability. This was indicated by ROC analysis and examination of the spatial classification accuracy against the realization. However, using the LST data alone did not provide a ROC curve that was significantly better than the sociodemographic rate data (the standard for examination). The utilization of only measures of LST did provide a model which only categorizes 12 census tracts in the lowest risk category which included a death. This aspect provides indication that potentially this particular model outperforms the selected sociodemographic measures of vulnerability.

All models utilizing LST showed that the most predictive variable was the mean temperature (LST) of the census tract. Maximum temperature was the second best performing LST variable, although it was inversely related to mortality. The interplay between mean and maximum LST suggests that the closer these values are to one another the greater the risk. If these measurements are close to one another and spatially coincident with areas of urban poverty, the elderly and/or the African-American population then the risk is even greater.

The results of this research have several practical implications for vulnerability modelling in planning and policy decisions concerning response to heat waves. Many cities do not incorporate information on where the most vulnerable populations reside as part of their mitigation planning. Emergency response professionals typically are aware of the locations of poorer neighbourhoods, but do not have the knowledge necessary to determine if these areas are coincident with areas of higher temperature. The process outlined in this work could be implemented to help close this information gap.

By using information on the spatial coincidence of vulnerability indicators during EHEs, warning systems and emergency response during such disasters could be directed in such a way as to save resources and foster their delivery in a timely manner. Maps from an alert system integrating social and environmental risk factors could be provided to emergency personnel, digitally or in analog form, so that mitigation activities could be directed in the field to the most vulnerable communities. The National Weather Service (NWS) has increased availability of sub-county level warnings for severe weather, such as tornados and severe thunderstorms, which inform both warning and response processes. Similarly, maps developed from processes presented in this paper could be used by local authorities to alert the public to where the intensity of the heat wave is most severe and to plan for the delivery of services to mitigate impacts in the most vulnerable communities

It is important to emphasize that the methods presented in this paper represent a first step toward developing a system for improving determination of risk to EHEs within a city. Several limitations should be noted. The remotely sensed processes used are also likely to introduce some uncertainties. The Landsat TM data used in this study provides a spatial resolution in the thermal channel of 120 m. This means that each pixel covers an area of 14,400 square meters. Since we are dealing with a highly urbanized area, many different land cover types are present, ranging from grass to impervious surfaces. Therefore, results are dependent on the spatial resolution of the imaging system. There is also error involved in the calculation of LST, much of which is due to atmospheric conditions at the time of image acquisition. Studies have suggested that typically this error is < 1 K [47]. In future studies it will likely be important to use a sensor of better thermal resolution (ASTER) so that error in LST estimation can be further diminished.

It has also been demonstrated that heat-related mortality is likely underreported due to the nature of identifying heat-related death and the lack of surveillance [11]. For example, a medical examiner performing an autopsy may identify a decedent's cause of death to be from a myocardial infarction. Another medical examiner may identify the same conditions from another decedent as being a myocardial infarction catalyzed by hyperthermia. Local individual level conditions play an important role. For example, some individuals living in high risk areas may have adequate protection from the extreme heat, such as air conditioning. Further, some vulnerable individuals may still have a very strong social network where friends or relatives can provide assistance when needed. The lack of social networks in elderly individuals has been shown to be a major contributor to heat-related death [48,49]. This type of individual level preparedness is impossible to measure without extensive person-to-person interviews.

Methods used in this study demonstrate a parsimonious approach to vulnerability mapping during extreme heat events. However, the transferability of the approach used in this study could be refined and validated using other retrospective datasets from extreme heat events in other locations. Also, other census variables could be examined in the context of vulnerability. It might prove beneficial to do an exploratory data analysis of a large range of other census variables to highlight which might either directly or indirectly elucidate vulnerability. Additionally, examination of thermal measures derived from other remote sensing platforms with greater spatial resolution should be explored.

Results of this research suggest that augmenting sociodemographic vulnerability with LST variables enhances vulnerability prediction during an extreme heat event. As with the remote sensing variables, examination of sociodemographic variables at finer spatial scales (e.g., block groups and neighbourhoods) should be explored; these finer levels of aggregation may yield different results. A goal of future studies in this area should be to inform policy and intervention during extreme heat events so that cities can better respond to this aspect of climate change impact on human health.

## Methods

In the summer of 1993, Philadelphia, PA experienced an EHE which lasted from July 3^rd ^to July 14^th^. The daily high temperature ranged from 35 to 38.5°C; with the low never below 23.3°C. A total of 118 deaths were directly attributed to this event [50-52]. Death certificates attributed to the heat event were collected from the Pennsylvania Department of Health and the addresses of the decedents were geocoded to current street centerline data for Philadelphia with 96.6% accuracy. Locations of death were then assigned to their respective census tracts (1990) with the output being a binary dataset; those census tracts with a heat-related death and those without.

Following studies already conducted on vulnerability to extreme heat, sociodemographic risk factors were extracted from the 1990 Census summary file 3 dataset for the 357 census tracts in Philadelphia [26,27,29,53]. These variables (shown in table 3) included population counts of Hispanic, African-American, Asian, Native American, other race, age 65 and over, age 65 and over in poverty, age 5 and under, persons below poverty and adults without a high school education. These data were added to the census tract dataset including the mortality data as both counts and rates of vulnerable groups per 1000 (1000/total population * number of persons in the vulnerable group).

**Table 3:** Descriptive statistics of the environmental and sociodemographic variables

**Variable**	**N**	**Minimum**	**Maximum**	**Mean**	**Std. Deviation**
*African Am. Rate*	357	0.0	1.0	0.4	0.4

*Native Am. Rate*	357	0.0	0.0	0.0	0.0

*Asian Rate*	357	0.0	0.8	0.0	0.1

*Other Race Rate*	357	0.0	0.7	0.0	0.1

*Hispanic Rate*	357	0.0	0.8	0.1	0.1

*Age 65 + Rate*	357	0.0	0.6	0.2	0.1

*Age 5 - Rate*	357	0.0	0.3	0.0	0.0

*Less than H.S. Ed. Rate*	357	0.0	0.6	0.2	0.1

*Poverty 65+ Rate*	357	0.0	0.2	0.0	0.0

*Poverty Rate (persons)*	357	0.0	0.8	0.2	0.2

*Total Population*	356	14.0	17971.0	4441.2	2936.0

*African Am.*	356	0.0	12154.0	1774.3	2347.2

*Native Am.*	356	0.0	110.0	9.3	15.4

*Asian*	356	0.0	1714.0	120.8	237.5

*Other Race*	356	0.0	7126.0	161.9	629.6

*Hispanic*	356	0.0	8798.0	235.7	769.8

*Age 65 +*	356	0.0	2949.0	677.5	548.0

*Age 5 -*	356	0.0	872.0	194.7	150.8

*Less than H.S. Ed.*	356	0.0	4822.0	1026.9	808.2

*Poverty 65 +*	356	0.0	573.0	106.5	98.6

*Poverty*	356	0.0	7262.0	880.3	913.3

*Population Density sqkm*	356	3.59	2908.2	879.3	466.9

*Minimum LST*	356	286.0	307.6	300.8	3.0

*Maximum LST*	356	301.9	316.1	308.7	1.8

*Range LST*	356	2.1	21.1	7.8	2.8

*Mean LST*	356	297.3	309.4	305.4	2.1

Population density (see table 3) is another variable which is suggested to be useful in the determination of vulnerability to extreme heat [26,54]. Studies have also suggested that population density and the intensity of the UHI are highly correlated, with R values exceeding 0.90 [55]. This relationship is often examined at the scale of the entire city. However, some studies have examined this relationship at finer scales and found that the positive relationship still exists [55-57]. Due to this apparent intrinsic relationship, population density was investigated as a potential explanatory variable in the analysis. We included a measure of total population for use with count-level data, and population density (total population/residential area) in the sociodemographic rate dataset.

In order to extract thermal characteristics a Landsat TM 5 scene for the Philadelphia region collected on July 10^th^, toward the end of the event, was acquired for processing. Landsat revisit times average 16 days so the collection of another image during this event was not possible. The dataset was spatially clipped using the minimum bounding rectangle for the Philadelphia county boundary. The spatial resolution of the thermal band for Landsat TM is 120 meters, sufficient enough to measure intra-census tract level variations in estimated land surface temperature (LST). LST is not directly equivalent to ambient air temperature which is measured by ground based thermometers (the standard high and low temperature in weather forecasts). LST is a remote measure of the thermal inertia of surface characteristics in the city. Ambient air temperature (collected *in situ*) measures the thermal inertia of the surface atmospheric components (i.e. air temperature). Previous studies have suggested that areas of higher surface temperature contribute to higher levels of localized ambient air temperature [36] and contribute to a decrease in human thermal comfort [33]. However, much uncertainty exists as to the exact relationship between surface temperatures and the ambient air temperature, which is dependent of wind conditions which are many times highly variable in urban areas. Much of this uncertainty has to do with urban geometry and land use land cover characteristics [31,37]. Wind also causes mixing in the atmosphere contributing to a decrease in ambient air temperature [36]. The present study assumes that the surface temperature contributes to a decrease in human thermal comfort during EHEs.

**Figure 4:**
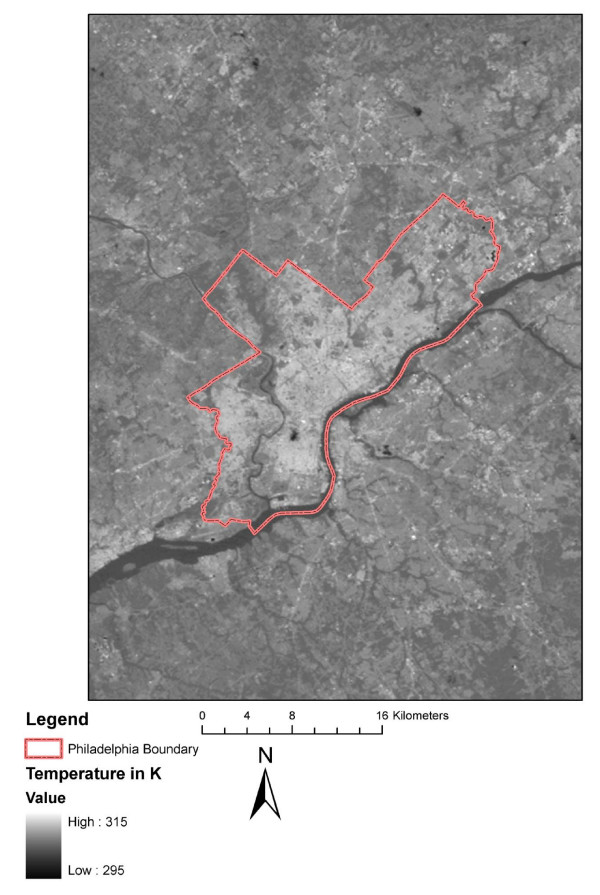
**Landsat TM Image of Philadelphia, PA, July 10, 1993**.

LST was estimated using the measure for at-satellite brightness temperature [34,58]. This method requires the input of the high and low gain of the sensor at the time of acquisition; in this case 11:00 am. After input the image values are converted into estimates of the LST in degrees Kelvin. One then can observe the relative values of the temperatures within the scene and can query pixels for a range of temperatures (Figure 4). A zonal calculation was done using the LST image and the census tracts (residential space within) of Philadelphia as the zone dataset. This created the minimum, maximum, mean, range, and standard deviation of LST (table 3) within each census tract (see section below on the calculation of temperatures within the residential space). The minimum and maximum are the values for the lowest temperature pixel and the highest temperature pixel respectively within the tract. The mean, range, and standard deviation of the LST utilize all pixels within the tract. These descriptive values are used as the temperature variables for comparison with mortality and sociodemographic values.

In order to facilitate spatially focused analysis it was necessary to calculate the amount and locations of residential space within all census tracts for Philadelphia during the study period. To accomplish this, the National Land Cover Dataset (NLCD) was collected for Philadelphia. The NLCD was developed from satellite imagery obtained during 1993 and thus is contemporaneous with the heat wave event. NLCD pixels containing high, medium and low density residential areas were re-coded into a single residential class. The thermal data that spatially coincided with residential areas within each census tract were used to calculate descriptive statistics of the thermal properties of the residential space. This was deemed an important step in the analysis of the thermal conditions of the residential areas. For example, if the thermal pixels contained in the entire census tract were used for the calculation of the thermal properties of the neighbourhood it could provide an erroneous characterization in the analysis. If a particular census tract under investigation contained an area of elevated surface temperature which was not coincident with residential space its inclusion would likely skew the results. Therefore, this step focuses the analysis on the thermal properties in areas where people reside. The minimum, maximum, mean, range and standard deviation of LST in residential areas were then added to the dataset containing the mortality and census sociodemographic data.

The primary method of analysis employed in this study is binary logistic regression. Logistic regression has been utilized in many epidemiological studies applying both aspatial and spatial methods [59-61]. All variables calculated for each census tract, were standardized by z scores and then examined in relation to the occurrence or non-occurrence of death from extreme heat. This provides a parsimonious indicator of the census tract properties which appear to be risk factors for mortality from the EHE. Five logistic regression models for predicting the occurrence of heat related mortality were compared: a model containing only census sociodemographic count data (model 1), one with LST data (model 2), another with sociodemographic count data supplemented with LST (model 3), one with census sociodemographic rate data (model 4; the standard to which the other models will be tested), and census sociodemographic rate data supplemented with LST (model 5). Model 4 is used to compare the other models for significance since sociodemographic rate data is so pronounced in the vulnerability literature.

All variables were assessed for multicollinearity by examining the variance inflation factors in multivariate linear regression. If the variance inflation factor exceeded a value of ten (10) then the variable was assumed to contribute significantly to multicollinearity and was removed from further analysis. After the removal of variables contributing to multicollinearity, the models were developed by adding all variables into an initial model. The variables which were not significant using the likelihood ratio F test (p < .05) were removed one by one until a model was found which included only variables which were statistically significant. Once we found variables that were significant we again tested those variables for multicollinearity using the variance inflation factors. The contributions of these remaining variables were assessed using Wald's test of significance (p < .05). The final five models were assessed for validity using the Hosmer-Lemeshow test at the .05 significance level.

Two final steps were used to further assess the validity of each model. First, Receiver Operator Characteristic (ROC) curves were created for each model. The models supplemented with LST were analyzed using this approach to determine if they significantly aided the classification (over using sociodemographic vulnerability alone) at the .05 significance level. The standard (model) used for comparison utilized sociodemographic vulnerability rate data which is typical of vulnerability models and is informed from previous studies [43]. The ROC method, suitable for binary response models, also allows for the direct visual and statistical comparison of the sensitivity of the model, provided such a model is statistically significant [61-64]. Performance of the models using the ROC method, are determined using the metrics of specificity and sensitivity. Sensitivity measures the proportion of actual positives, in this case census tracts containing a heat-related death, correctly identified. The higher the value for sensitivity the more likely all census tracts containing a death are correctly tagged. It is calculated by (number of true positives/number of true positives + number of false negatives). Alternately, specificity measures the proportion of negatives which are correctly classified, (number of true negatives/number of true negatives + number of false positives). The higher the specificity the fewer the false positives; census tracts not containing a death labelled as containing a heat-related mortality.

Secondly, the odds of each census tract having an occurrence of death, using each of the 5 models, were added to a census tract level dataset and stratified using a quartiles approach. This classified each census tract into the range of, 0-25%, 26% - 50%, 51% - 75%, 76% - 100%, probabilities of the occurrence of death.

## Competing interests

The authors declare that they have no competing interests.

## Authors' contributions

DPJ: Devised the study using logistic regression, extracted the data, performed the analysis and wrote the rough copy of the manuscript

JSW: Aided in the final construction of the manuscript and further examined the scientific merit of the study

GCL: Aided in the final construction of the manuscript (especially the background section) and further examined the scientific merit of the study.

All authors have examined and approved the final manuscript.
